# Reports of the Glasgow Asylum for Lunatics

**Published:** 1824-03-01

**Authors:** 


					1824] ( 7/5 )
III.
Annual Reports of the Directors of the Glasgow Asylum
for Lunatics, for the years 1814, 1815, 1816,1817, 1818,
1819, 1820, 1821, and 1822.
Glasgow has long been distinguished among the great cities
of the world for the extent, and wise discriminations of her
munificence. Her institutions,* established for the purposes
-of mitigating the evils of misfortune and diease, are nume-
rous, and all of them excellent; but, above the rest, her asy-
lum for the reception of lunatics constitutes a prominent ob-
ject of attention to those who venerate that most generous of
virtues?the active charity which^rejoices in labouring to al-
leviate misery determined by the alienations of mind.
Knowing the constitution of this Asylum to bean approved
model which the patrons of other houses for the insane have
found advantage in imitating, we propose submitting to our
readers whatever in the Reports of its Directors we shall dpem
conducive to the illustration of its particular economy. Our
present article, however, is not intended to be exclusively
a contribution to the pathological philosophy of insanity; it
is rather to be regarded as a sketch in medical topography,
which may be useful in enabling subsequent inquirers to ap-
preciate the comparative results of treatment on the manifes-
tations of intellectual disease.
Our plan, therefore, will lead us?1st, to exhibit, in a tabu-
lar form, the annual number of persons treated in this Asylum;
2d, to give a synoptical classification of the cases into male
and female, old and recent, together with a view of the dif-
ferent gradations of the malady; and 3d, to select such por-
tions of the Reports, ordinations, as may appear subservient
to the design in which we are engaged.
I. The Asylum for Lunatics at Glasgow comprehends 136
apartments, destined for the accommodation of 120 patients,
who are distributed into classes according to their sex and
sphere of life, and according to the degree of their insanity.
A similar distinction is observed in the grounds for exercise
and airing, to which each class is separately admitted.
* For a full account of these the reader is referred to the Typogra-
phical Picture of Glasgow, with a Sketch of a Tour to the Highlands, and
Falls of Clyde, 12mo, with plates. Glasgow, Printed for Richard Griffin
and Co. 1820.
Vol. 17. No. 16. 5 G
776 Analytical Reviews. [March
Paupers from Glasgow, or from such towns and parishes
as have contributed 50/. to the Institution for every 1500 of
their inhabitants, may be placed in the Asylum at the weekly
rate of 8s. and all others at that of 10s. 6d. for each indivi-
dual. Persons in the middle and higher ranks of life are re-
ceived as boarders, on terms varying from 15s. to 63s. per
week, for which they obtain suitable lodging and attendance.
After having surmounted many difficulties, the Directors of
this Asylum, towards the conclusion of 1814, had so far com-
pleted their manifold operations as to be enabled to declare
the house ready for admission of patients. At their request,
therefore, and introductively to its being opened, the magis-
trates of Glasgow, on December 1st of that year, inspected
it; and, after minute examination, publicly announced their
entire satisfaction with every part of the establishment. On
the 12th, twenty-three men and eighteen women were trans-
ferred to it from the cells in the town's hospital. Four of
the men, however, appearing to be idiots, were remitted. One
female and two males were subsequently received ; and thus,
at the end of the year, the house contained forty lunatics.
Nine annual reports of their transactions have been pub-
lished by the directors, with the laudable object of submitting
the progressive state of the Asylum to general observation.
In the subjoined table, originally constructed by Mr. Drury,
the superintendant of the Institution, and inserted in the
Eighth Report, is rspresented the yearly summary of persons
admitted into the Asylum, together with a view of their ulti-
mate disposal.
Males. Females. Total.
Amitted ...in 1814  21 19 40
181 5  39 37 76
181 6  52 33 85
181 7  44 40 84
181 8  50 40 * 90
181 9  43 34 77
182 0  51 32 83
182 1  56 37 93
182 2  50 30 80
406  302  -708
Re-admitted from Relapse,, in 1816...... 4 2 6
1817...... 1 1 2
181 9  2 2 4
182 0  3 1 4
182 1  0 1 1
1822  1 0 1
?  11   7   18
Total of Admissions       417 309 726
1824J Reports of the Glasgow Asylum for Lunatics. 777
SDtemtestfD.
Males. Females. Total.
Dismissed Cured    in 1815  16 16 32
181 6  24 16 40
1JB17  26 17 43
181 8  15 21 36
181 9  21 18 39
182 0  31 13 44
182 1  21 14 35
182 2  26 7 33
180  122  302
Dismissed Relieved in 1815  2 6 8
1816  10 11 21
181 7  13 14 27
181 8  14 15 29
181 9  6 6 12
182 0  7 13 20
182 1  15 12 27
182 2  12 1 3 25
79   90  169-
Dismissed by desire in 1816  1 1 2
181 7  4 1 5
181 8  8 7 15
181 9  7 6 13
1820.  5 3 8
182 1  5 6 11
182 2  13 12 25
43   36   79
Dismissed Unfit... in 1815  2 0 2
1816  1 0 1
1818  2 13
181 9  3 0 3
182 0  1 0 1
182 1  1 0 I
182 2  0 0 O
Died..   in 1815  1 0 J
181 6  3 2 5
181 7  2 1 3
181 8  5 3 8
181 9  6 4 10'
182 0  5 3 8
182 1  9 2 11
1822  11 2 13
42 ? 17  r 59
Total of Dismissions, and of Deaths  354 266 620
Remaining.. ..31st December, 1822  63 43 106
417 309 726
II. Introductively to their tabular classification of patients,
which commences in the Third Report, the Directors explain
its objects and use in the following terms
" In order to begin a series of documents, which, if carefully con-
tinued, may tend to establish some conclusions at present doubtful,
and to correct others erroneous, a list extracted from the Records by
the superintendent, with great care and.labour,.is subjoined. The
778 Analytical Reviews. [March
terms used are more conformable to the language of our law than
to the specific distinction of nosology. The imbecile, the fatuous,
and idiots, graduate into each other, differing chiefly in degree. The
first are weak in body as well as mind; the second, though some-
times robust, are more stupid, and the third are more stupid still.
By old cases, are meant those that have continued beyond one year;
the average in the list being from five to eight years : recent cases,
are those under one year's duration."
Each of the last seven Reports contains four tabular sketches
intitled, Male Patients, Old Cases ; Male Patients,
Recent Cases; Female Patients, Old Cases; Female
Patients, Recent Cases ; exhibiting a complete list of per-
sons admitted into the Asylum, from the period of its opening
on December 12th, 1814, to June 1st, 1822; and, at the
same time, shewing the changes that have taken place in each
of those remaining, together with the state of them, on leav-
ing the house, who were dismissed, within that time. We
have taken the labour of concentrating the general results of
these 28 tables into the two following; which, we trust, to
many of our readers, will not be devoid of interest.
No. 1.?MALE PATIENTS.
Classification of
the Cases when
admitted.
Furious.
Melancholy...
OLD CASES.
How
Dismissed.
Sure of
those re-
ning
Furious and Me-
lancholy
Imbecile.......
Fatuous
Idiots
Total
12
12
10
10
32
16
> s
5.2
43
19
10
8
39
24
148
RECENT CASES.
How
Dismissed.
U
83
24
128
45
18
14
26
u -
?.2
5 ?
h E
147
52
14
16
23
1
253
1824] Reports of the Glasgow Asylum for Lunatics. 779
No. 2.?FEMALE PATIENTS.
Classification of]
[the Cases when
admitted.
Furious
Melancholy....
Furious and Me
lancholy
(imbecile ...
Fatuous....
Idiots
[Total
OLD CASES.
RECENT CASES.
How
Dismissed.
.1 'I
"3 I >?
Pi,?
el i
121
9
0
28 ICl
State of
those re-
maining
24
14
10
33
CO
o 5?
e3 u
2 ?
r* u
42
20
How
Dismissed
35
1
110
47
35
98
14
14
34' 1G'
State ofj
those re-
maining
? c
** o
QJ .JS
U3 +??
o> g
4-1
2 J
ce y
14!
83
64
13
17
182
Note. In the. general Table the total number of patients admitted into
the Asylum is 726; in these two Tables of Classification the number is
only 693, making a difference of 33, which is accounted for by the former
Table being made to contain all the admissions for the whole of 1822,
whereas the latter Tables do not include those admitted subsequent to the
last day of June in that year.
From these lists it appears; 1 st. That the males exceed the
females in number, as 391 to 292, or nearly as four to three,
a disproportion not usual in other Asylums ; 2nd. That, in
both sexes, the disease becomes less and less curable in pro-
portion to its duration ; out of 258 old cases, 34 cures only
\vere accomplished, whereas no less than 226 out of 435
recent ones were cured : hence appears the necessity of
?placing insane persons as early as possible under proper
treatment; 3d. That the relative curability of male and
female lunatics, is very uncertain, and not materially diffe-
rent : out of 391 males, there were 150 cures, and 110 out
of 292 females; and 4th. That a continued series of such
780 Analytical Revieivs. [March
lists, will furnish data for comparing the success of different
institutions with each other; but, for this purpose, say the
Directors, the peculiarities of each must be fairly stated : for
example, many of the largest Asylums in Britain, after the
trial of one year, regularly dismiss all the patients not cured^
at that time ; but here, with a few exceptions, the incurables
continue accumulating from year to year, and persons are
admitted under every form and complication of the disease,
and even though apparently near death.
III. We are now to complete the last section of our
Article, by placing before the readers of this Journal, a
miscellaneous selection of observations taken from the nine
Reports, according to the order in which they were originally
submitted to general inspection.
A. 1814.?This Report is solely occupied with statements
relating to the civil economy of the Asylum, and the ad-
mission of its first inmates, in the last month of the year.
B. 1815.?Here, we find some useful suggestions on the
employment and office of keepers. The system of locking
up patients for hours together, or of putting them in chains
whenever the keepers cannot attend, has no place in this in-
stitution. Under such a system, an asylum puts on the ap-
pearance of a jail, instead of being a dwelling of compara-
tive comfort. No person is confined here, but for a fault, or
to prevent mischief, when any one shews signs of becoming
outrageous. To find keepers properly qualified and to keep
them so, has always been and always will be the most diffi-
cult part of the very difficult task allotted to those who manage
an Asylum. Besides activity and strength, perfect sobriety
and unremitting vigilance, a keeper should have a quick ap-
prehension to discern the first approach of a paroxysm, great
decision, and the greatest humanity. Such qualities, how-
ever, do not often meet in one character; and, when they
do, their possessor can generally find a station equally lucra-
tive, and more agreeable than the irksome attendance on the
insane. Besides the weariness of confinement, his temper is
put to the most severe trials. He is frequently exposed to
personal assaults ; every peculiarity about him is mimicked ;
his features and dress are caricatured on the walls; he is
often assailed with the most cutting sarcasms and virulent re-
proaches, which seem prompted by malignity of heart more
than want of understanding. Hence, he requires the greatest
self-command to suppress resentment, and to refrain from
punishing those who are so much in his power, and where
lie has so many chances of taking revenge without detection.
The unhappy lunatic is generally reckoned undeserving of
1824] Reports of the Glasgow Asylum for Lunatics. 781
credit: having been found guilty of innumerable exaggera-
tions and much falsehood, he is not believed even when he
speaks the truth ; whence, under the very eye of men of much
penetration, of strict honour, and great humanity, the most
shocking abuses have, in some cases, continued for years with-
out being discerned.
With reference to the permanency of the cure of lunatics,
the subsequent remarks occur. It is certain, that all are
liable to relapse, just as one who has had an ague this spring,
is liable to relapse next autumn; but this is no good reason
for saying that agues cannot be cured, or that it is idle to
make the attempt. It is certain, also, that many are ready
to relapse after stated intervals, unless particular care be
taken to break the morbid habit, and to avoid the exciting
causes of derangement; but it is no less certain that many,
long subject to derangement, have continued free from it for
many years, and that the number of complete permanent
cures has, of late, increased greatly, under mild treatment,
especially in houses where it is not the interest of any one to
prolong the residence of the patient.
C. 1816.?In this report, we meet with some thoughts on
a subject, in the discussion of which much ingenious spe-
culation might be exercised. It is to be regretted, that the
eloquent author of the paper (Dr. Cleghorn) has declined
favouring us with his own views on this intricate subject.
It is difficult to apprehend, we are told, and more difficult to
explain, the principle on which the demolition of furniture
should give ease to the mind of a madman ; for, all the
while, he knows a chair and a table to be what they are, and
he knows also that they belong to one who supports him
with much difficulty, and who, far from being able to re-
place what he destroys, can hardly keep lip the present
scanty stock. On some occasions, indeed, reasons quite
absurd, but somewhat intelligible, are given for such an out-
rage. Some, becoming suddenly frantic, break to pieces
every thing that can be broken in their apartments, because
they think the articles might be much better made ; others,
because they deem it not only allowable, but commendable,
to encourage trade in unpropitious times. As the love of
strong drink can be restrained only by keeping it out of the
madman's reach, so the propensity to breaking cannot, in
many cases, be restrained without a degree of confinement
hardly compatible with health.
D. 1817.?Some observations occur in this paper, on the
effects of drugs, when administered to the insane, and on the
loathsome practice of forcing aliment into their stomachs.
Among others, we find the statement, that it has been ob-
782 Analytical Reviews. [Marek
served, with emphasis, by some reporters of diseases in towns
and dispensaries, that the reports of most institutions are
searched in vain for medical information. Reports like this,
however, are quite unfit for medical disquisitions ; but the
time may yet arrive when facts ascertained in this Asylum,
together with the practice found most successful there, and
the inferences which should be drawn from the facts and the
practice, will be laid before the public* in a proper form.?
The difficulty of ascertaining the real effects of drugs is great
in every disease, and particularly so in cases of insanity, on
account of its peculiar nature. In the opinion of the best
judges, medicines have been given improperly in this, as
often as in any malady ; and they have got the credit of cures
which they seem to have had no share in producing. There
is, indeed, great reason to suspect that, in many instances,
cures have been ascribed to drugs which, though prescribed
by the physician, and carefully furnished by the apothecary,
were never tasted by the patient. There are, no doubt,
some persons, both in mad-houses and out of them, who
think themselves neglected if they do not get regularly a
portion of drugs : to all these, medicine ought to be promptly
furnished. Others dislike it so much that they refuse not it
only, but for fear of it their food also : to such, therefore, no
medicine is given except on the greatest emergency, and
then concealment of it in their food, or persuasion has
always ensured its exhibition. As to the horrid custom of
forcing it, as well as food, down their throats, under which
some have been choked completely, others thrown in con-
vulsions, and all more injured by the violence used, and by
their own furious opposition, than they could possibly be
benefited by the operation of any drug whatever, it (forcing)
has never been permitted in this Asylum.
E. IS 18.?We are told here, that it has been said, " none
but a madman would reason with a madman;"?a most
equivocal maxim, oftener founded on apathy, indifference,
* We understand this statement to have come from the pen of Dr.
Robert Cleghorn, who, at the time, was physician to the Asylum. This
highly gifted individual preceded Dr. Thomas Thomson in the Chemical
ChairHbf:* tlie University of Glasgow, and stood unrivalled at the head of
Session in the West of Scotland, for many years before his death.
It,'matter of profound regret, that he did not live to complete and
publish these half-promised observations on the treatment of insane
plfSbns'. "He was endowed with great learning and eloquence; possessed
an of^ti&l* philosophic, and discriminative mind; and, from very ex-
tensive experience, as well as profound reflection, was every way quali-
fied to illustrate any subject in pathology, which he might have chosen
to make the object of his research.
1824J Reports of the Glasgow Asylum for Lunatics. 783
arid carelessness, than on superior discernment. In fact, as
men of sound mind are occasionally less under the influence
of motives than they ought to be ; so the least sound, while
short of idiotcy, still feel the influence of motives in no in-
considerable degree, and it is impossible to fix with precision
the point, at which motives lose their influence so completely
as not to be worth urging. A long chain of reasoning is,
no doubt, out of the question ; but a short argument, a sen-
tentious maxim gravely stated, or an aphorism delivered
with solemnity, especially if in the language of scripture,
often produces a sudden calm amid the most furious com-
motions, and leaves durable effects. When a religious me-
lancholy madman was loudly avowing and vindicating his
intention to commit suicide, the very first opportunity, his
hand was taken kindly, and in a solemn tone he was reminded
of the awful consequences of such a deed, 'c as no murderer
hath eternal lifeon which his eyes, formerly glaring with
phrenzy and despair, were turned towards the ground ; he
appeared absorbed in fixed thought, and made no more
mention of suicide. This man afterwards declared, with
much emotion, that whenever the temptation recurred, the
text always came along with it, uniformly shielding him from
its baneful effects.
F. 1819.?The following hints relative to the medical
economy of the Glasgow Asylum are found in this report.
The treatment of the patients has been varied according to
the features and the causes of their lunacy.?Internal reme-
dies have, in a few instances, been of great service; but, in
general, medicine has been of little avail. The warm bath,
sometimes conjoined with the affusion of cold water on the
shaven head, has been much used, and often with advantage.
Some patients have derived benefit from the cold bath.
Exercise, especially in the open air, has been of general
utility, and much good has been done, in several cases, by
pretty severe bodily labour. Rotatory motion, by means
of a whirling chair, has of late been tried in a great number
of cases, and, in some of them, with wonderfully good effects.
By the contrivance used for this purpose, the degree and
duration of the motion are completely under command. The
operation is always performed in the presence of the phy-
sician, and in no instance has it been followed by any of
those formidable symptoms, which have sometimes occur-
redin other institutions, where a more simple, but much less
manageable, apparatus was employed. In some instances of
unusual docility, benefit has been obtained from attention to
certain rules of mental discipline. But the moral manage-
ment of the patients, by the usual means of restraining their
Vol. IV. No. 16. 5 H
784 Analytical Reviews, [March
Tiolence, of correcting their evil habits and propensities, and
of regulating their conduct and behaviour, lias been of more
general utility than any other practice employed. These
means, however, are often found to be of little avail, unless
those by whom they are used, besides gaining complete as-
cendancy over the patient, gain, at the same time, his con-
iidence and good will.?One of the frantic patients, by for-
cibly striking his head against the wall of his apartment,
had a part of his scalp torn up, so as to expose to view a
large portion of his skull. He lost a great quantity of blood;
but, by proper treatment, the wound readily healed. It is
remarkable, that after this accident,* the state of the patient's
mind gradually improved, and, in the course of two months,
he was dismissed perfectly cured. Another frantic patient
accidentally received a similar wound, and soon after, in like
manner, recovered.
When outrage is committed, the discipline of the Asylum
requires that the offender should be put under restraint.
But this is done with the least possible severity, and with
due attention to health and comfort. The keepers are under
the strictest rules, insomuch, that none dare confine a patient
without an order of the physician or superintendant; and
they are prohibited from using opprobrious, or even pas-
sionate language, in the discharge of their duty. Striking,
beating, or in any way maltreating the patients, is totally
inadmissable: any keeper found guilty of such cruelty
would be instantly dismissed, perhaps otherwise punished,
according to the enormity of his offence. For the purpose
of rendering any refractory patient more docile, the securing
of his hands or confining him to his apartment, is the means
usually employed, and is seldom found to be long necessary.
During favourable weather, the patients are placed in the
airing grounds occasionally, and almost all of them daily;
and, sociality is often promoted, while the irksomeness of
confinement is alleviated, by various occupations and amuse-
ments.
* From this fact, two deductive suggestions seem naturally to present
themselves to the inquirer's mind : 1st. That the patient's insanity was
not a primary affection, but the consecutive result of morbid action in
one of the encephalic organs; 2nd. That counter-irritation and san-
guineous depletion phanged this morbid action, relieved the lesioned
organ, and ultimately terminated the man's intellectual malady, by re-
balancing those cerebral functions, from derangement of which hij
lunacy did evidently proceed. It may, therefore, be matter of useful
investigation, to determine the manifestations of mental disease, on
which an imitative substitute for this accidental treatment shall operate
with equal success.
1824] Reports of the Glasgoiv Asylum for Lunatics. 78$
G. 1820.?This Report informs us that, in regard to the
treatment of lunacy, much still remains obscure. In many
cases, little good can be done, but it is of importance to
know enough, if possible, not to do harm ; and, in ordcc
to acquire this knowledge, some experience is necessary.
Many patients have been judiciously treated before being
sent to the Asylum ; but some, even of considerable stand-
ing, have not been at all under medical care, or, being placed
at inconvenient distances from their proper medical attend-
ants, have been committed chiefly to the care of persons not
sufficiently skilled in the treatment of the disease. V iolent
measures are still too often employed, where milder, judi-
ciously conducted, would be more effectual. Powerful drugs,
also, and irritating applications, are not unfrequently ad-
ministered without due discrimination. Some cases do not
admit of great evacuations, and are even aggravated or ren-
dered more obstinate by depletion ; yet, to the inexperienced,
these cases may appear to be similar to others, where copious
bleedings and other evacuations are indispensably necessary.
This opinion of the Glasgow physician is corroborated by
that of Pinel, Esquirol, and other eminent writers, all of
whom are sparing of the lancet, and lay much stress on the
necessity of carefully distinguishing those cases where bleed-
ing may be useful from those in which it must be injurious.
After some remarks in recommendation of treating the
insane with mildness, the Reporter proceeds to submit some
hints on their moral and mental management. Among other
means for promoting this object, the attendance of lunatics
on divine service is mentioned, and its advantages discussed.
Dr. Burrows, who bestows high encomiums on this Asylum,
is of opinion, that religious consolation, imparted privately,
would be preferable to public worship; and it may be
so, says the Report, in particular cases, and especially if
the service were performed agreeably to some established
modes. For example, were it performed according to the
mode of the Church of England, where each individual
of the congregation repeats and responds at certain parts of
the service, it might perhaps lead to an unbridled license of
the tongue, which would, indeed, as Dr. B.says, be a mockery
of religion. But the form of worship, according to the
Church of Scotland, is not liable to the same objection ; and,
as to preaching to unselected auditors, on topics calculated
to excite strong emotions or to foster gloomy impressions, we
admit that it would be highly injudicious. Yet, with due
respect for the opinion of the author, we cannot, adds the
Glasgow physician, approve of the mode which he prefers.
If a religious monitor were to enter the wards of a lunatic
786 Analytical Review*. [March
asylum, he would unavoidably be assailed by patients with
whom it was not intended that be should hold any communi-
cation. The very persons, moreover, to whom his attention
might be expressly directed, would be injuriously chagrined
or irritated, unless they were indulged in expatiating on the
very subjects, on which their minds were already too prone
to dwell. But all speculative opinion must yield to the
evidence of facts derived from actual experience. Sermons
have been preached in this Asylum, to numerous and most
attentive audiences, for more than four years, and have not
yet been observed to produce any injurious effect on the
mind of any one of the auditors. Some of the patients, it
is true, may have listened to these sermons without having
been much edified, but all of them have been gratified, and
many of them soothed and consoled.
These sketches are followed by others, on the influence of
laborious employment in promoting the cure of lunacy.
Some patients, it is said, whose cases appeared to be almost
hopeless, recovered by labouring in the airing grounds. On
various occasions, when this employment was for a time sus-
pended, some of the working patients, whose state of mind
had been rapidly improving, remained without farther amend-
ment, or even grew worse; but soon after labour was re-
sumed, they again proceeded to improve. In one remarka-
ble instance, in which the most fanciful and gloomy antici-
pations predominated, whenever the patient was warmed by
toil, his mind was relieved. In general, so speedy was the
favourable change in this case, that it seemed as if the vapours
of the brain exhaled with the sweat of the brow ! The bene-
ficial effect of labour on the mind is twofold : it serves not
only to dissipate gloomy and incoherent thought by day,
but also to prevent the distressing illusions of the night, by
preparing the patient for sound repose.
- Many of the lunatics, we are informed, write letters which
are correctly and even elegantly penned. A great proportion
of these are addressed to the physician, while others, not
proper to be despatched, are preserved ; because the letter
of a patient serves as an indication, and as a record of the
state of his mind, and may also prove to be a useful docu-
ment, if his case should ever afterwards become the subject
of legal investigation. The object of the writer often is to
demand the reason of his confinement, or to prove that he
ought to be instantly liberated. The style of some is diffuse
and rhetorical; others reason more closely ; and one inge-
nious person, whose literary productions are extremely cu-
rious, occasionally employs the form of syllogism. Some
patients exhibit, in their letters, a wonderful degree of con-
1834"! Reports of the Glasgow Asylum for Lunatics. 787
sistency and acuteness. It is still, perhaps, too Common to
suppose that all lunatics are, in point of mental degradation,
nearly equal; and, therefore, that they are all equally re-
moved from a capacity for common feeling and from a right
to common sympathy. But the forms of lunacy are so vari-
ous, and aberration from soundness of mind is, in some cases,
so slight, that it would be no easy task to determine the pre-
cise bounds of rationality.
H. 1821.?Mild treatment and exercise, in the form of
recreation or labour, are again stated in this Report as hav-
ing been found eminently beneficial in the management of
lunatics. Many of them occupy their thoughts in writing
essays, letters, poems, &c. One of our patients, it is said,
lias written an ingenious vindication of himself from the im-
putation of lunacy. He philosophically observes," I find it
difficult to divest the mind of recollections on the topics of
business, especially in the situation in which I am placed ;
for the mind naturally reverts to such subjects as have been
familiar to it in times past, on which it exerts its energies in
multiplying ideas and conceptions, for lack of reasonable oc-
cupation ; and, in a maze and labyrinth of conjecture, seeks
to find out the cause of being immured with beings in the
most lamentable state of existence." Another of these pati-
ents, in a disquisition on lunacy, distributes the different per-
sons affected with it into three classes:?Dementes, or those
who are deficient in mental capacity; Vacui, or those who
are completely void of ideas ; and the Pleni, or those who
possess high ideas of themselves and of their worldly riches
and grandeur?lords of the universe?possessors of superna-
tural powers?divinely inspired?hearers of the heavenly
choristers?and receivers of celestial visions and annuncia-
tions.
In madness, says the Report, the power of reasoning on
some subjects may remain ; but when called to act in oppo-
sition to the predominant illusions, it proves to be but too
feeble an antagonist for those workings of the imagination.
One of the surest signs of amendment is an admission by the
patient, of fallacy in any of his former illusions; ana, in
cases approaching to convalescence, it is often highly interest-
ing to observe the reasoning faculty thus beginning to resume
its wonted ascendancy. Such improvement of the mind is
sometimes very naturally accompanied with diffidence of judg-
ment. Instead of immuring the insane, without distinction,
in the depressing gloom of dismal apartments, where they
are deprived of all the accommodations to which they have
been accustomed, we should endeavour, says the reporter, to
afford them every comfort likely to soothe their troubled spi-
788 Analy tical Reviews. [March
rits; especially as the enjoyment of such comforts is often
one of the best means of promoting their recovery.
This Report returns to the advantages of preaching sermons
to the insane. The experience of all those clergymen who
have hitherto preached, must have convinced them that the
patients selected to be their auditors were capable of listening
attentively, and of behaving in the most quiet and orderly
manner. Yet, some persons still doubt the propriety of
preaching in the Asylum. If, indeed, all our patients were,
at all times and on every subject, insane from the time when
they were admitted until the instant of their dismission, the
attempt to impart to them religious instruction might prove
to be fruitless, if not injurious. But some persons are insane
only on certain points; some are insane only at certain times,
but enjoy long intervals of reason; some are in a state of con-
valescence from insanity; and, although not possessed of suf-
ficient firmness of mind, nor so free from occasional aberra-
tion, as to render it expedient for them to resume their former
occupations, or even to mingle with the world, yet are per-
fectly capable of deriving benefit from moral and religious
instruction ; and, lastly, some are not lunatics, in any degree,
but completely cured of that lunacy for which they were ad-
mitted; yet, according to the rules of this Asylum, they are
obliged to remain in it for two months, or, in some cases of
periodical madness, for a longer period, as the necessary
term of their probation. The illusions of the insane, no
doubt, are sometimes derived from religious impressions, and
then great discretion is requisite in regard to religious exer-
cises. But we do not find, among our patients, that religion
is a frequent cause of lunacy; and many of our convalescents
or probationers, whose malady had not any connexion with
religion, are much disposed to attend to religious duties, and
to cherish those impressions which they received in their
early years, from a religious education. Such persons, be-
fore the practice of preaching in the Asylum was introduced,
often complained bitterly that they were precluded front at-
tending divine service; and sometimes, at their own earnest
entreaties, they were indulged with liberty to go to church,
under the charge of a proper attendant. This indulgence
was, for obvious reasons, objectionable; but, surely, it would
be hard to withhold from such patients the gratification of
that public worship, through which our excellent clergymen
now humanely impart to them useful admonition and conso-
lation.
I. 1822.?We extract the following observations from the
Ninth Report; they will not be without their usefulness.
The following detail of symptoms, derived from our obser-
1824] Reports of the Glasgow Asylum for Lunatics. 7^9
vation, or consistent with our experience, say the reporters,
may serve, without the aid of professional knowledge, to di-
rect attention to the state of the mind on the approach of lu-
nacy, and when proper treatment is likely to be of great avail.
The first indications of lunacy are various; and, as might
be expected in the accounts given of a malady which exhibits
a great diversity of forms, they have been variously described.
Head-ache, giddiness, throbbing of the temples, or impaired
vision, have ushered in a paroxysm; and hypochondriacal
apprehensions, arising from a disordered state of the diges-
tive organs, have terminated in maniacal illusions. In many
instances, the symptoms first remarked, are a defect in the
power of attention, fits of absence, frequent talking or mut-
tering of the patient to himself, unmeaning fixed staring of
the eyes, a dejected countenance, and sometimes jerking mo-
tions of the body, or odd gesticulations. Together with theso
appearances, the mind is perhaps under the depressing influ-
ence of hurt pride, disappointed hope, or religious apprehen-
sions ; perhaps it is brooding over some feeling of remorse,
fear,, jealousy, or chagrin, on grounds which are wholly ima-
ginary. Love is, in some instances, the predominant im-
pression ; and it is equally singular and characteristic, that
the object of this affection and the patient are sometimes
unacquainted with each other. The first indication, in some
patients, is an extraordinary flow of high spirits, about to
end, at length, in maniacal delirium ; in others, extreme
terror is first noticed. The countenance is pale, ghastly, and
strongly expressive of the inward emotion; the speech is hur-
ried and tremulous; and the extremities are cold, perhaps
bedewed with a cold sweat. Soon, however, the eye glares
malignantly, the face flushes, and assumes the expression of
ferocity, the objects of terror become the objects of vengeance,
and the patient is furious. In some, there is an unusual de-
gree of suspicion or of anticipation of evil, and a belief iri
imaginary plots or conspiracies. In others, there is great
irascibility and malignity, and some act of desperation, ven-
geance, or cruelty, is perhaps the first obvious symptom of
die malady. From the commencement of lunacy, and espe-
cially as long as the mind continues to be in a state of excite-
ment, patients generally sleep little or none; yet some are
disposed to lie constantly in bed, and are unwilling to answer
questions, or to converse with their friends or relations. In
some instances, the patient carefully conceals his illusions for
a long time after they have taken possession of the mind.
Perhaps, for the first time, he reveals them confidentially to
his clergyman, or to his medical attendant. As soon, how-
ever, as maniacal illusions are betrayed, the nature of the
case is manifest.
790 Analytical Reviews. [March
The different physiological views which have been taken
of the nature of lunacy, the reporters go on to say, may have
possibly had some share in leading to certain discrepancies,
observed in the accounts given of the symptoms. Some of
the most intelligent writers on the subject are not even agreed
as to the organ which is primarily affected ; nor have they
settled whether lunacy is to be ascribed chiefly to moral or to
physical causes. We are disposed to think that the disease
originates in the brain; and that, perhaps, in every instance
there exists a physical cause or predisposition to insanity.
The active remedies which are frequently employed with be-
nefit, in cases of indigestion, may sometimes owe their good
effects, more to their influence on the state of the bead than
is generally supposed ; and, where there is a known predis-
position to lunacy, the possibility that a loss of tone in the
digestive organs may proceed from some change going on in
the brain itself, ought not to be overlooked.
From the preceding sketches may be elicited a general
idea of the mode, economical, moral, and medical, of treat-
ing insanity in the Asylum at Glasgow; and we trust, in con-
clusion, that to many of our readers, the information they
contain will be acceptable as well as useful.

				

