# Corrigenda in This Number

**Published:** 1824-03-01

**Authors:** 


CORRIGENDA IN THIS NUMBER.

Page 775, for *typographical*, read topographical.

